# Lumbar puncture-related cerebrospinal fluid leakage on magnetic resonance myelography: is it a clinically significant finding?

**DOI:** 10.1186/1471-2253-13-35

**Published:** 2013-10-27

**Authors:** Keita Sakurai, Noriyuki Matsukawa, Kenji Okita, Minoru Nishio, Masashi Shimohira, Yoshiyuki Ozawa, Susumu Kobayashi, Takemori Yamawaki, Yuta Shibamoto

**Affiliations:** 1 Department of Diagnostic Radiology, Tokyo Metropolitan Medical Center of Gerontology, 35-2 Sakaecho, Itabashi-ku, Tokyo 173-0015, Japan; 2 Department of Neurology and Neuroscience, Nagoya City University Graduate School of Medical Sciences, Nagoya, Japan; 3 Department of Neurosurgery, Nagoya City University Graduate School of Medical Sciences, Nagoya, Japan; 4 Department of Radiology, Nagoya City University Graduate School of Medical Sciences, Nagoya, Japan; 5 Department of Clinical Neuroscience and Therapeutics, Hiroshima University, Graduate School of Biomedical Sciences, Hiroshima, Japan

**Keywords:** Lumbar puncture, Cerebrospinal fluid leakage, Post-dural puncture headache, Magnetic resonance myelography, Magnetic resonance imaging

## Abstract

**Background:**

Post-dural puncture headache (PDPH) due to excessive cerebrospinal fluid (CSF) leakage is a well-known complication of lumbar puncture. Although various factors, especially the type of spinal needle, have been demonstrated to be associated with PDPH, the clinical implications of CSF leakage detected on magnetic resonance myelography (MRM) images remain unclear. The objective of this case–control study was to evaluate the association between radiologically visualized CSF leakage and PDPH.

**Methods:**

Clinical data including patients’ age and gender, types of spinal needle, duration of bed rest, interval between lumbar puncture procedures and MRM studies, and incidence of PDPH were compared between patients who were radiologically-positive and -negative for CSF leakage.

**Results:**

Of the 22 patients with definite CSF leakage on MRM images, most were asymptomatic (86%, 19/22). The remaining three patients, who were suffering from PDPH, only complained of headaches and were treated conservatively. In a review of patients’ clinical data, there were no significant differences in any parameter including the incidence of PDPH between the 22 patients who were radiologically-positive for CSF leakage and the 31 radiologically-negative patients.

**Conclusion:**

The significance of radiologically visualized CSF leakage should not be overestimated, as most such incidents are not associated with PDPH and do not require any treatment.

## Background

Lumbar puncture is generally performed in daily medical practice to measure the pressure in the subarachnoid space, to obtain cerebrospinal fluid (CSF) samples for analysis, to inject contrast medium for myelography, or to induce spinal anesthesia. However, puncturing the dura has the potential to lead to excessive CSF leakage, and CSF hypovolemia subsequent to excessive CSF leakage can lead to post-dural puncture headache (PDPH), which has been regarded as a complication of lumbar puncture for over a century [1]. Its clinical characteristics including its incidence and associated factors have been evaluated in previous studies [2,3].

Recently, neuroimaging techniques including radioisotope cisternography (RICG) and magnetic resonance imaging (MRI) have enabled the visualization of postpuncture CSF leakage in the epidural space [4,5]. Considering its pathophysiology, it is indisputable that postpuncture CSF leakage contributes to the development of PDPH [2,3]. However, the incidence and clinical implications of radiologically visualized postpuncture CSF leakage have rarely been evaluated [6,7]. These studies evaluated only a small number of selected subjects, so a larger number of unselected subjects in daily medical practice appear necessary for the evaluation of the clinical implication of radiologically visualized postpuncture CSF leakages. Furthermore, such iatrogenic CSF leakage can make it difficult to differentiate between PDPH and spontaneous intracranial hypotension (SIH) [4,5]. Taking these problems into consideration, we aimed to investigate the clinical implication of radiologically visualized postpuncture CSF leakage in daily medical practice.

## Methods

### Subjects

This was a retrospective study evaluating the incidence and clinical characteristics of postpuncture CSF leakage using data obtained at a single medical center, and was approved by the Ethics Committee for Clinical Research of Nagoya City University Graduate School of Medical Sciences, which waived the requirement for informed consent. The privacy of the patients was completely protected. Between January 2009 and March 2012, 329 lumbar punctures were performed to obtain samples for CSF analysis in 251 patients with various neurological disorders (e.g., multiple sclerosis, infectious meningitis, etc.) at the Department of Neurology. Of these 329 examinations, 270 were excluded because no subsequent magnetic resonance myelography (MRM) study was performed within 14 days of the lumbar puncture. There were two reasons why MRI examinations were not performed in these patients. The one was that their primary illness did not require lumbar MRI examinations (e.g. viral meningitis), and the other was that the examination and admission schedules did not allow to perform MRI examinations after lumbar punctures. As a result, 59 examinations involving 53 patients who underwent subsequent thoracolumbar or lumbar MRM studies were included in this study. The lumbar puncture procedures were mainly performed using 21-gauge (G) Quincke spinal needles at the L3/4 or L4/5 intervertebral level. PDPH was diagnosed according to the previously published diagnostic criteria [3].

### MRM protocol and image analysis

The thoracolumbar or lumbar MRM studies were performed on a 1.5-T imager (Gyroscan Intera; Philips Medical Systems, Best, The Netherlands) using a synergy spine phased-array coil. The 2D MRM sequence was performed using the following parameters: turbo spin echo (TSE) sequence; repetition time/echo time, 8000 ms/1000 ms; field of view, 350–500 mm; matrix, 512 × 142; slice thickness, 40–50 mm; section orientation, coronal; and TSE factor, 256. Basically, this sequence was utilized as a localizer scan in our institution. Postpuncture CSF leakage was diagnosed according to the previously reported imaging findings by the consensus of two experienced physicians (K.S. and M.N.), who were blinded to the patients’ clinical information [5,8]. Additionally, to differentiate CSF leakages from mistakable findings such as water component at the intervertebral joints, root sleeves and perineural cysts, postpuncture MRM findings were compared with findings of other sequences such as axial and sagittal T2-weighted images in each patient.

### Statistical analysis

Statistical analyses were carried out using the SPSS 11.0 statistical software program (Dr. SPSS II for Windows, standard version 11.0; SPSS Inc., Chicago, IL, USA). The unpaired *t* test was used for comparisons of age distributions. Fisher’s exact test was used for comparisons of the gender distribution and PDPH incidence between the two patient populations. Pearson’s chi-squared test was used for comparisons of the spinal needle gauge and puncture level. The Mann–Whitney U test was used to compare the duration of bed rest and the interval between the lumbar puncture and MRM study. Differences were considered significant when *p* < 0.05.

## Results

**Figure 1:**
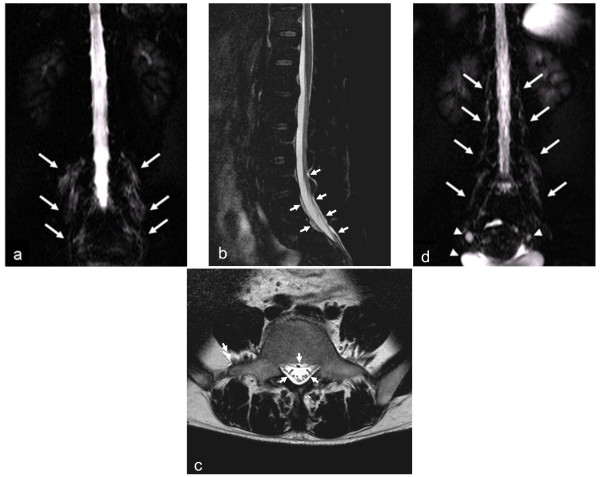
**Representative magnetic resonance myelography (MRM) images of postpuncture cerebrospinal fluid (CSF) leakage.** 2D MRM images were performed about 28 and 6 hours after the lumbar punctures in a 44-year-old male with chronic inflammatory demyelinating polyneuropathy (patient A) and a 42-year-old female with multiple sclerosis (patient B), respectively. Bilateral fluid collection around the nerve roots and paraspinal area **(a, d)** were depicted on 2D MRM images (arrows). Additionally, sagittal fat-suppressed T2-weighted **(b)** and axial T2-weighted **(c)** images of patient A revealed abnormal epidural and paraspinal fluid collections (arrowheads). In spite of such leakage, patient A was asymptomatic. However, patient B complained of an orthostatic headache that had persisted for six days. Arrowheads indicate fluid accumulation that was unrelated to CSF leakage (e.g., the bladder and ovarian cysts).

The characteristics of the patients are summarized in Table 1. Twenty-two MRM studies involving 22 patients exhibited postpuncture CSF leakage (37%, 22/59). All except four of these radiologically visualized leakages were bilateral and were mainly located in the paraspinal areas at the lumbosacral level (Figure 1). Of the 22 patients, only three suffered from PDPH (14%, 3/22), which occurred within 48 hours of the lumbar puncture and persisted for one to six days. No other symptoms associated with PDPH, such as nausea, vomiting, or hearing loss, were observed. These patients were treated in a conservative manner including bed rest, appropriate hydration, and non-steroidal anti-inflammatory drugs. The other asymptomatic patients did not require any kind of treatment for their CSF leakage.

**Table 1:** Patient characteristics

	**CSF leakage-positive exams (n = 22)**		**CSF leakage-negative exams (n = 37)**		**p value**
Age (years)	50 ± 18 (17–86)		56 ± 19 (17–86)		0.27
Gender (male: female)	12: 10		19: 12*		0.66
Underlying disorders					
Neuropathy/neuritis	8		6		
Demyelination	4		9		
Infection	3		2		
Hydrocephalus	1		3		
Degenerative disease	1		3		
Others	5		8		
Gauge of spinal needle	21 G	-19	21 G	– 18	0.18
	19 G	– 1	19 G	– 2	
	23 G	– 1	23 G	– 6	
	Unknown	– 1	Unknown	– 11	
Puncture level	L3/4	– 4	L3/4	– 6	0.69
	L4/5	– 12	L4/5	– 16	
	L5/S	– 0	L5/S	– 1	
	Unknown	– 6	Unknown	– 14	
Duration of bed rest (hour)	1 h	– 1	1 h	– 0	0.06
	1.5 h	– 1	1.5 h	– 0	
	2 h	– 20	2 h	– 37	
Duration between LP and MRM (days)	1.8 ± 2.6		4.0 ± 4.3		0.13
Post-dural puncture headache	3 (14%)		5 (14%)		0.66

Data are shown as absolute numbers or the mean ± standard deviation.

*Six of the CSF leakage-negative patients underwent two CSF analyses.

Note: CSF = cerebrospinal fluid; exams = examinations; n = number of exams; G = gauge; L = lumbar; S = sacral; h = hours; LP = lumbar puncture; MRM = magnetic resonance myelography.

In 37 CSF leakage-negative MRM studies, five patients suffered from PDPH (14%, 5/37). In a review of the patients’ clinical data including the incidence of PDPH, their basic characteristics and the details of puncture procedures, no significant differences were detected in any parameter between the radiologically CSF leakage-positive and -negative patients (Table 1).

## Discussion

Considering the advantage of high contrast resolution, ability to depict the entire spinal subarachnoid space including fluid collections and leakages, and non-invasive nature (i.e., no LP and no radiation exposures), MRM may be regarded as the first-line examination in the diagnosis of CSF leakages. Its high contrast resolution contributes to detect indirect findings such as epidural-paraspinal fluid collections, especially small amounts of leakages along bone structures [9-11]. Furthermore, MRM with intrathecal gadolinium injection can provide both physiologic and morphologic information, which enables the detection of direct CSF leakages with higher sensitivity than any other technique including computed tomography myelography [9]. In the present study, similar to the previous study evaluating ICSFL on MRM [5], CSF leakage was distributed around nerve roots and paraspinal area at the lumbosacral level. Predominant lumbosacral distribution was not surprising because the thecal punctures were performed at this location. It is expected that these characteristic paraspinal fluid collections are the result of CSF escaping from the epidural space into the paraspinal loose connective tissues, similar to the retrospinal C1–2 fluid collection reported in patients with PDPH and SIH [12,13]. In addition to these anatomical factors, the low resolution of the 2D MRM sequence depicts CSF leakages in the paraspinal area more definitely than those around nerve roots in this study.

Although the association between PDPH and CSF abnormalities (i.e., between CSF loss and a reduction in intracranial pressure) is not disputed, the exact pathophysiology of PDPH remains unclear. PDPH is considered to be caused by the hole left in the dura after the lumbar puncture needle has been withdrawn, which can allow CSF leakage from the subarachnoid space [14]. Among the various risk factors for PDPH including puncture procedure variables, patient characteristics, and a past history of chronic headaches, the size and design of the needle used for the lumbar puncture are the most significant determinants of PDPH [2,3,15]. As a result, its incidence can vary widely, depending on the population involved and the needles and techniques used [3,16,17]. The incidence of PDPH in this study (14%) was comparable to that described in a previous report in which 20G cutting spinal needles were used [18].

It is worth noting that the incidence of PDPH did not differ significantly between the radiologically CSF leakage-positive and -negative patients in our study. On the surface, these findings do not seem to support the hypothesis that CSF leakage through dural holes causes PDPH. However, several previous studies have indicated that the volume of CSF lost via leakage and CSF hypotension are not associated with PDPH and have also questioned the dural hole hypothesis [16,19]. In addition, neuroimaging studies performed with MRI or RICG after lumbar puncture have revealed that some patients with postpuncture CSF leakage are asymptomatic [4-6]. However, such leakages can still cause clinical problems, especially with the diagnosis of disorders such as SIH [5]. Together, these findings indicate that the existence and volume of CSF leakage are not necessarily associated with PDPH and suggest that the underlying mechanisms of PDPH are more complex.

A simple older explanatory model for PDPH is that the reduction in intracranial pressure induced by persistent CSF leakage causes traction between pain-sensitive structures such as meningeal membranes, blood vessels, and nerves [3,20]. On the other hand, hypersensitivity to substance P and the Monro-Kellie doctrine, which suggests that compensatory intracranial vasodilatation is induced by CSF leakage, have recently been recognized as viable hypotheses regarding the cause of PDPH [1,21]. Considering these new hypotheses, it is not surprising that some of the patients in this study without definite CSF leakage on MRM images complained of PDPH.

A number of limitations of the present study need to be addressed. The main limitation is the relatively small study population. The lack of available clinical data due to the study’s retrospective nature is also problematic point. These made it difficult to evaluate various factors that affect the incidence of PDPH, such as the number of lumbar punctures, body mass index, and the patients’ medical histories. Furthermore, the incidence of CSF leakages on MRM might also be affected by the number of lumbar punctures. Another limitation is our use of the 2D MRM sequence; i.e., the lower resolution made this study qualitative rather than quantitative evaluation. To perform the precise measurement of CSF leakage, it is necessary to use the 3D MRM sequence, which achieves higher spatial resolution. Additionally, the interval between lumbar punctures and MRM may be too long in some cases. Therefore, there is a chance of resorption of CSF leak in radiologically-negative patients. In spite of these limitations, the fact remains that most of the patients with definite CSF leakage after lumbar puncture with the relatively large 21G Quincke spinal needle were asymptomatic.

## Conclusion

In this study, most radiologically visualized CSF leakages are not associated with PDPH and do not require any treatment. It is important that we gain a better understanding of this asymptomatic and incidental phenomenon in order to avoid misdiagnosis and overtreatment.

## Competing interests

The Authors declare that there is no conflict of interests.

## Authors’ contributions

KS drafted the manuscript. KS and MN performed the neuroimaging analysis. NM, KO and TY participated in the design of the study and evaluated the clinical information. MS and YO performed the statistical analysis. SK helped to check the imaging protocol. YS participated in its design and coordination and helped to draft the manuscript. All authors read and approved the final manuscript.

## Pre-publication history

The pre-publication history for this paper can be accessed here:

http://www.biomedcentral.com/1471-2253/13/35/prepub
